# Perspective of obstetric care‐providers on being involved in cervical cancer screening during antenatal care in the Netherlands

**DOI:** 10.1002/cam4.7380

**Published:** 2024-07-05

**Authors:** Nick M. A. van der Hoeven, A. J. C. van den Brule, H. J. van Beekhuizen, I. M. C. M. de Kok, F. J. van Kemenade

**Affiliations:** ^1^ Department of Gynaecological Oncology University Medical Centre Rotterdam Rotterdam the Netherlands; ^2^ Department of Pathology Jeroen Bosch Hospital 's‐Hertogenbosch the Netherlands; ^3^ Department of Public Health, Erasmus MC University Medical Centre Rotterdam Rotterdam the Netherlands; ^4^ Department of Pathology University Medical Centre Rotterdam Rotterdam the Netherlands

**Keywords:** cancer prevention, community outreach, gynecological oncology, screening, women's cancer

## Abstract

**Background:**

The aim of this study was to determine attitude of Dutch midwifes, gynecologists and general practitioners (GPs) towards involvement in antenatal cervical cancer screening (CCS) in the Netherlands.

**Methods:**

In 2021, Dutch midwives, gynecologists, and GPs were offered a single digital questionnaire assessing perceived feasibility, benefits, and harms of antenatal CCS.

**Results:**

A total of 6943 Questionnaires were send and response rate was 18% (*N* = 1260). Of all respondents, 78% considered antenatal CCS via obstetric care providers feasible. Most respondents (85%) agreed that offering CCS in person can increase motivation to attend. Most midwives (93%) considered that women would feel less encumbered if cervical sampling would be performed by obstetric care providers, rather than by GPs.

**Conclusion:**

Results indicate that introduction of antenatal CCS is considered feasible by a majority of Dutch midwifes, gynecologists, and GPs. Considered benefits include improved motivation to attend and reduced test related barriers.

## Graphical abstract

Dutch healthcare professionals involved in obstetric care consider introduction of cervical cancer screening via obstetric care feasible.

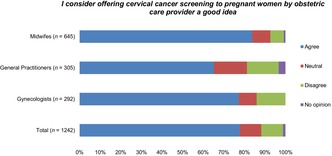

## 1 INTRODUCTION

Low attendance hampers effectiveness of Dutch cervical cancer screening programme (CCS). The Dutch CCS starts at the age of 30, and is primarily high‐risk human papillomavirus (hrHPV) based.^1^ At the time of this study, women were given a possibility of receiving a self‐sample device, but only after no show, the default being cervical sampling at their general practitioners (GP) office. Lowest participation (<50%) is observed amongst youngest women aged 30–40 years, especially those from urbanized areas with low socio‐economic status, and ethnic minorities, driven by various logistic, motivational, and test‐related barriers.^2^, ^3^, ^4^ In other countries with national CCS programme such as England, better uptake of screening and subsequent cancer protection is observed, highlighting the importance of increasing attendance in Dutch CCS programme.^5^

Informing about CCS and conducting screening is currently not part of standard Dutch obstetric care, mainly due to limited reliability of historical cytology‐based CCS in pregnancy. However, current knowledge on cervical cancer highlights the causative role of hrHPV, being reflected both in form of vaccine‐based therapies, adoptive T‐cell therapy and immune‐modulating agents in patients with advanced cervical cancer, as well as preventive strategies including hrHPV vaccination and hrHPV‐based CCS.^6^ With introduction of Dutch primary hrHPV based screening in 2017 therefore, the earlier mentioned limitation of cytology‐based screening is no longer applicable in theory. Dutch obstetric care is easily assessable and free of charge. Most women undergo obstetric care at a near home first‐line midwifery practice and a smaller fraction will attend a hospital, the latter under supervision of a gynecologist.^7^ If un‐ and under screened women would be selected and personally informed by familiar and trusted obstetric healthcare providers, a part of logistic, motivational, and test‐related barriers to attend CCS might be circumvented, possibly increasing attendance.

Little is known about opinion of obstetric care providers to support such a policy of giving them a role in CCS programme. Via this questionnaire study, we assessed (differences in) perceived feasibility of antenatal CCS amongst Dutch midwifes, gynecologists and GPs, including potential benefits and harms.

## 2 METHODS

This study was conducted between April 2021 and December 2021. We offered a single questionnaire digitally to Dutch gynecologists, GPs, and midwifes, including associated residents or trainees (Appendices [Link], [Link], [Link]). Respondents were asked about their personal and professional background, and their opinion on introduction of antenatal CCS in the Netherlands. The questionnaire single‐ or multiple‐choice questions, 6‐point array questions with predefined statements on antenatal CCS, with answer options in the latter ranging from ‘totally disagree’ to ‘totally agree’, including ‘no opinion’, and a single open ended question assessing respondents age. None of the questions or statements were obligatory to answer. No reminders were send and all responders had 3 months to submit their answers.

We invited professionals in various ways; (1) using the mailing list the Dutch gynecologist professional organization (2) using the mailing list of Dutch regional university departments of general practice. (3) Contacting individual general practitioners practices via email using a web‐based registry, with the request to distribute the questionnaire amongst all GPs affiliated to the practice, estimating the total amount of GPs receiving a questionnaire based on a nationwide average of 2.4 GPs per practice.^8^, ^9^ contacting individual midwifery practices via telephone using a web‐based registry, with the request to distribute the questionnaire amongst the stated number of midwives affiliated to the practice.^10^

### 2.1 Statistical analysis

The primary outcome was the percentage of respondents who considered antenatal CCS via obstetric care provider feasible. Secondary outcomes were the percentages of respondents (dis)agreeing with each statement and differences between midwifes, gynecologists, and GPs. For array questions, we grouped answers in four groups, namely a group ‘(dis)agree’ if a respondent provided the answer ‘totally (dis)agree’ or ‘(dis)agree’, a group ‘neutral’ and a group ‘no opinion’. Frequencies and percentages were calculated for discrete variables and means and ranges were calculated for respondent’ age. Differences between and in groups of midwifes, gynecologists, and GPs were compared using the chi squared test for discrete variables. A *p <* 0.05 was considered significant. The statistical analysis was carried out using SPPS version 28 (IBM Corp. Released 2021. IBM SPSS Statistics for Windows, Version 28.0. Armonk, NY: IBM Corp).

## 3 RESULTS

Table 1 shows the number of professionals receiving a questionnaire, response rates and background information of respondents. In total, 6943 care providers received a questionnaire and 1260 (18.1%) responded. Here amongst were 1538 gynecologists, 2996 GPs, and 2409 midwifes receiving a questionnaire and 294 (19.1%), 312 (10.4%), and 654 (27.1%) responding, respectively. Additional background information of respondents can be found in Appendix S4.

**TABLE 1:** Total number or responders, response rates and background information about responders by profession.

	GPs	Gynecologists	Midwives	Total
Professionals receiving questionnaire (*N*)	2996	1538	2409	6943
Professionals providing a response (*N*)	312	294	654	1260
Response rate (%)	10.4	19.1	27.1	18.1
Number of residents or trainees amongst respondents (*N* (%))	71 (22.8)	65 (22.1)	16 (2.4)	152 (12.1)
Average age (mean, range)	43.2 (21–67)	44.5 (27–68)	36.7 (20–68)	40.3 (21–68)
I provide obstetric care weekly (*N* (%))	1 (0.3)	118 (40.1)	654 (100%)	773 (61.3)
I perform colposcopy (*N* (%))	n.a.	104 (35.4)	n.a.	n.a.
I perform colposcopy in pregnant women (*N* (%)	n.a.	48 (16.3)	n.a.	n.a.
I treat women with cervical Cancer (*N* (%)	n.a.	26 (8.8)	n.a.	n.a.

**FIGURE 1:**
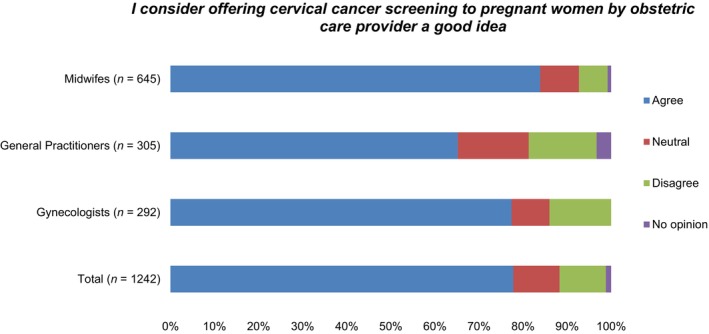
Considered feasibility of antenatal CCS via obstetric care providers.Chi‐squared test: GPs versus gynecologists *p* = 0.01; GPs versus midwifes *p* < 0.001; gynecologists versus midwifes *p* = 0.001.

The opinion of healthcare providers on feasibility of CCS via obstetric care providers is shown in Figure 1. In total, 77.8% of all respondents considered antenatal CCS via obstetric care providers feasible. Significantly more midwifes (83.9%) considered antenatal CCS via obstetric care provider feasible, when compared both to gynecologists (77.4%; *p* = 0.001), and GPs (65.2%; *p* < 0.001).

Outcomes of answers provided to all other statements are provided in Appendices S5 and S6. Of all favorable statements, healthcare providers agreed most with the statement: ‘*I* *suppose* *women can be motivated more to attend CCS by obstetric care provider in person,* *rather than via anonymous leaflets*’. Midwifes agreed significantly more with this statement (90.6%), compared both to gynecologists (83.7%; *p* = 0.001), and GPs (74.4%; *p* < 0.001). Also, significantly more midwives (92.2%) agreed with the statement: ‘*I suppose women are less encumbered by cervical sampling if performed by obstetric care provider rather than general practitioner*’, when compared both to gynecologists (46.3%; *p* < 0.001), and GPs (25.9%; *p* < 0.001). Less than half of midwifes considered themselves skilled in cervical sampling (49.7%) or familiar with current CCS guidelines (47.0%).

## 4 DISCUSSION

This study demonstrated willingness amongst obstetric care providers to become more involved in cervical screening in the Netherlands, hitherto not being part of the screening programme. Main advantages of antenatal CCS considered by all respondents were better motivation in person and, with some variation dependent on the professional background, sampling for screening via obstetric care as a reduced burden compared to sampling by GP.

Pregnant women in the Netherlands attend an average of 12 consultations during pregnancy, regardless their social background, and these consultations are experienced as respectful and confidential.^11^, ^12^, ^13^ We therefore assume that routine obstetric care provides many opportunities to personally check woman's CCS attendance and motivate un‐ or under screened women to participate. Nearly all midwifes are female, contrary to GPs, and due to gender preferences and familiarity with internal investigations by obstetric care provider, resistance against cervical sampling if performed by the latter may possibly be lower compared to GP conducted sampling.^3^, ^14^

### 4.1 Strengths

We were able to contact an estimated 6943 (37%) of 1500 gynecologists, 3900 midwifes, and 13.500 GPs registered in The Netherlands.^8^, ^14^ Overall response in our study was 18% and the response rate of gynecologists and midwifes was 22% and 27%, respectively, being comparable to other Dutch questionnaire studies for both groups of professionals.^15^, ^16^ Distribution of age, province(s) of residence, work‐area(s), and practice size amongst responding midwifes and GPs (Appendix S4) was comparable to nationwide registries, indicating representative sampling.^8^, ^14^

### 4.2 Limitations

Response of GPs in our study was lower (10%), compared to other Dutch questionnaire studies, with response rates of 20%–30% in the latter.^17^, ^18^ Variances in study design, a possible overestimation of the amount of GPs receiving a questionnaire and contacting practices via unannounced email in our study may explain these differences. We used a short, non‐validated questionnaire, so we consider results in our study more as a rough estimation of professional opinion rather than a thorough and highly sensitive assessment. Furthermore, other stakeholders in antenatal CCS and/or obstetric care we not included.

Based on results of this study, a crucial next step would be to further evaluate the potential harms and benefits of antenatal CCS in the Netherlands. This may be done both via practical pilot studies in which CCS is actually counseled and offered via obstetric care providers, but also theoretically, using mathematical modeling to determine long‐term (cost‐) effectiveness of adding antenatal CCS to the Dutch screening program.^19^

If antenatal CCS is considered to be effective, logistical, and organizational issues need to be addressed and tested prior to implementation. A framework enabling obstetric care providers to counsel pregnant women effectively has to be established, since only half of midwifes in our study considered themselves familiar with CCS guidelines. Optimal timing of CCS counseling and sampling should be subject to debate. First, a substantial amount of antenatal screening is already offered in first‐ or second trimester, and prevented must be to overload women with screening tests in this period.^20^ Second, abnormalities found at CCS during pregnancy would require all pregnant women undergoing first‐line care to be referred to second‐line care for further evaluation. Third, CCS participation may cause anxiety or distress and such negative emotions could be worse and longer lasting in case further evaluation of abnormal screening results is hampered because of pregnancy.^21^ Alternatively, we consider that counseling may be performed *during* pregnancy, whilst sampling is not conducted until *after delivery*, for example at routine post‐partum check‐up, circumventing some of the here above described drawbacks.

## 5 CONCLUSION

Our study found that the majority of professionals involved in Dutch CCS and/or obstetric care were willing to consider combining antenatal care with cervical screening in the Netherlands feasible. Considered advantages of antenatal CCS by respondents include motivational benefits and reduced test‐related barriers. Prior to introduction, research should establish efficacy of antenatal CCS, potential harms of antenatal CCS as well as required changes to current CCS framework and guidelines.

## AUTHOR CONTRIBUTIONS

**Nick M. A. van der Hoeven:** Conceptualization (equal); formal analysis (equal); investigation (equal); writing – original draft (equal). **A. J. C. van den Brule:** Writing – review and editing (equal). **H. J. van Beekhuizen:** Conceptualization (equal); writing – review and editing (equal). **I. M. C. M. de Kok:** Writing – review and editing (equal). **F. J. van Kemenade:** Conceptualization (equal); writing – review and editing (equal).

## FUNDING INFORMATION

This research did not receive any specific grant from funding agencies in the public, commercial, or not‐for‐profit sectors.

## ETHICS STATEMENT

Patients were not involved in this study. The study was exempt from institutional review board approval because data were gathered and analyzed anonymously.

## Supporting information


Appendix S1.

Appendix S2.

Appendix S3.

Appendix S4.

Appendix S5.

Appendix S6.

## Data Availability

The data that support the findings of this study are available from the corresponding author upon reasonable request.

## References

[1] National Institute for Public Health and the Environment (RIVM) . Framework for the Execution of Cervical Cancer Population Screening 2017.

[2] Netherlands Comprehensive Cancer Organisation (IKNL) . Monitor National Cervical cancer screening programme. 2022.

[3] Bosgraaf RP , Ketelaars PJ , Verhoef VM , et al. Reasons for non‐attendance to cervical screening and preferences for HPV self‐sampling in Dutch women. Prev Med. 2014;64:108‐113. 24736093 10.1016/j.ypmed.2014.04.011

[4] Bongaerts TH , Buchner FL , Middelkoop BJ , Guicherit OR , Numans ME . Determinants of (non‐) attendance at the Dutch cancer screening programmes: a systematic review. J Med Screen. 2020;27(3):121‐129. 31801039 10.1177/0969141319887996 PMC7491249

[5] Choi S , Ismail AA‐O , Pappas‐Gogos GA‐O , Boussios SA‐O . HPV and Cervical Cancer: A Review of Epidemiology and Screening Uptake in the UK. Pathogens. 2023;12(2):298 10.3390/pathogens12020298 36839570 PMC9960303

[6] McLachlan J , Boussios S , Okines A , et al. The impact of systemic therapy beyond first‐line treatment for advanced cervical cancer. Clin Oncol (R Coll Radiol). 2017;29(3):153‐160. 27838135 10.1016/j.clon.2016.10.002

[7] Perined . annual report. 2022. https://assets.perined.nl/docs/a5d79719‐1320‐4d7c‐8191‐d1d573852982.pdf

[8] Flinterman L , Vis E , de Geit E , Batenburg R . Netherlands Institute for Health Services Research. 2021. Nivel Registration of Professional General Practitioners. https://www.nivel.nl/sites/default/files/bestanden/1004234.pdf 2021.

[9] Netherlands Institute for Health Services Research . Find a general practitioners practice near your home address. 2021 https://www.kiesuwhuisarts.nl/

[10] Royal Dutch Association of Midwifes . Find a midwife near your home address. 2021. https://deverloskundige.nl/zoek‐een‐verloskundige

[11] Dutch Society of Obstetrics and Gynecology . Guideline Basic Prenatal Care: Diagnostics of Main Pregnancy Complications in Low Risk Pregnancies (second‐ and third‐line care). 2015.

[12] Chote AA , de Groot CJ , Bruijnzeels MA , et al. Ethnic differences in antenatal care use in a large multi‐ethnic urban population in The Netherlands. Midwifery. 2011;27(1):36‐41. 19939527 10.1016/j.midw.2009.07.008

[13] van der Pijl MSG , Kasperink M , Hollander MH , Verhoeven C , Kingma E , de Jonge A . Client‐care provider interaction during labour and birth as experienced by women: respect, communication, confidentiality and autonomy. PLoS One. 2021;16(2):e0246697. 33577594 10.1371/journal.pone.0246697 PMC7880498

[14] Kenens R , Batenburg R , Netherlands Institute for Health Services Research . Nivel Registration of Professional Midwifes. 2021. https://www.nivel.nl/sites/default/files/bestanden/1004097.pdf

[15] bij de Weg JM , Visser L , Oudijk MA , de Vries JIP , de Groot CJM , de Boer MA . Improved implementation of aspirin in pregnancy among Dutch gynecologists: surveys in 2016 and 2021. PLoS One. 2022;17(6):e0268673. 35679244 10.1371/journal.pone.0268673 PMC9182337

[16] Cronie D , Perdok H , Verhoeven C , et al. Are midwives in The Netherlands satisfied with their jobs? A systematic examination of satisfaction levels among hospital and primary‐care midwives in The Netherlands. BMC Health Serv Res. 2019;19(1):832. 31722747 10.1186/s12913-019-4454-x PMC6854733

[17] Plat FM , Peters YAS , Giesen P , Smits M . Availability of Dutch general practitioners for after‐hours palliative care. J Palliat Care. 2018;33(3):182‐186. 29607714 10.1177/0825859718766947

[18] Bakker L , Kemper PF , Wagner C , Delwel GO , de Bruijne MC . A baseline assessment by healthcare professionals of Dutch pharmacotherapeutic care for the elderly with polypharmacy. Eur J Pub Health. 2017;27(4):679‐686. 28637234 10.1093/eurpub/ckx076

[19] Jansen E , Naber SK , Aitken CA , de Koning HJ , van Ballegooijen M , de Kok I . Cost‐effectiveness of HPV‐based cervical screening based on first year results in The Netherlands: a modelling study. BJOG. 2021;128(3):573‐582. 32638462 10.1111/1471-0528.16400 PMC7818441

[20] National Institute for Public Health and the Environment . Policy framework for Prenatal and Neonatal Screening (2018–0043). 2018.

[21] Stonehocker J . Cervical cancer screening in pregnancy. Obstet Gynecol Clin N Am. 2013;40(2):269‐282. 10.1016/j.ogc.2013.03.005 23732031

