# Supplementary material for: Perspective of obstetric care‐providers on being involved in cervical cancer screening during antenatal care in the Netherlands
Source: Cancer Med. 2024 Jul 5;13(13):e7380. doi: 10.1002/cam4.7380 (PMC11224965; doi:10.1002/cam4.7380)
Supplement: Supplementary file 1 — Appendix S1. [file CAM4-13-e7380-s002.docx]

**Appendix A:** questionnaire provided to gynecologists and residents

| **Personal information** | | | | | | | | | | | | | | | | | | | | | | | |
| --- | --- | --- | --- | --- | --- | --- | --- | --- | --- | --- | --- | --- | --- | --- | --- | --- | --- | --- | --- | --- | --- | --- | --- |
| 1 | Current age | | | | … years | | | | | | | | | | | | | | | | | | |
| 2 | Are you employed currently? | | | |  Yes, as a resident in gynecology   Yes, as a gynecologist   Yes, but as a resident in gynecology nor as a gynecologist   No | | | | | | | | | | | | | | | | | | |
| 3 | If a resident, in which year of your training are you currently? | | | |  First   Second   Third | | | | | | | | | |  Fourth   Fifth   Six | | | | | | | | |
| 4 | In which province(s) are you working currently? | | | |  Drenthe   Flevoland   Friesland   Gelderland | | | |  Groningen   Limburg   Noord-Brabant   Noord-Holland | | | | | | | | | | |  Overijssel   Utrecht   Zeeland   Zuid-Holland | | | |
| 5 | In which affiliation(s) are you working currently? | | | |  General Hospital   University Hospital   Private Clinic | | | | | | | | | | | | | | | | | | |
| 6 | What is your professional scope as a gynecologists or resident? | | | |  Perinatology   Oncology   Urogynecology | | | | | | | | | |  Benign Gynecology   Reproductive Medicine   None | | | | | | | | |
| **Your experience with obstetric care** | | | | | | | | | | | | | | | | | | | | | | | |
| 7 | I provide obstetric care weekly |  Yes | | | | | | | | | | |  No  *Please go to question 11* | | | | | | | | | | |
|  |  | Totally disagree | | | Disagree | | | | | | | Neutral | | | Agree | | | | | | Totally agree | | |
| 8 | I always question CCS participation at pregnancy intake |  | | |  | | | | | | |  | | |  | | | | | |  | | |
| 9 | I always provide information on CCS to stated non-responders |  | | |  | | | | | | |  | | |  | | | | | |  | | |
| 10 | Pregnant women ask me whether I can perform CCS during pregnancy |   *Please go to question 12* | | |   *Please go to question 12* | | | | | | |   *Please go to question 12* | | |  | | | | | |  | | |
| 11 | I perform CCS, if pregnant women ask me |  | | |  | | | | | | |  | | |  | | | | | |  | | |
| 12 | I perform colposcopy on a weekly base |  Yes | | | | | | | | | | | | |  No  *Please go to question 15* | | | | | | | | |
| 13 | I perform colposcopy in pregnant women often |  Yes | | | | | | | | | | | | |  No  *Please go to question 15* | | | | | | | | |
| 14 | I am involved in treatment of women with cervical cancer on a weekly base |  Yes | | | | | | | | | | | | |  No | | | | | | | | |
| **Positive statements on antenatal CCS** | | | | | | | | | | | | | | | | | | | | | | | |
|  |  | | | | Totally disagree | | Disagree | | | | Neutral | | | | | Agree | | | Totally agree | | | | Totally disagree |
| 15 | I consider CCS during pregnancy via attended obstetric care provider feasible | | | |  | |  | | | |  | | | | |  | | |  | | | |  |
| 16 | I consider CCS during pregnancy via general practitioner feasible | | | |  | |  | | | |  | | | | |  | | |  | | | |  |
| 17 | I consider CCS of non-pregnant women via first-line midwives feasible | | | |  | |  | | | |  | | | | |  | | |  | | | |  |
| 18 | I suppose CCS is more easy to schedule during pregnancy, for a majority of women | | | |  | |  | | | |  | | | | |  | | |  | | | |  |
| 19 | I suppose women can be motivated more to attend CCS by obstetric care provider in person, rather than via anonymous leaflets | | | |  | |  | | | |  | | | | |  | | |  | | | |  |
| 20 | I suppose women are less encumbered by cervical sampling if performed by obstetric care provider rather than general practitioner | | | |  | |  | | | |  | | | | |  | | |  | | | |  |
| 21 | I think that most first-line midwives are skilled in performing cervical sampling | | | |  | |  | | | |  | | | | |  | | |  | | | |  |
| 22 | I think that most first-line midwives are well known with current CCS-programme | | | |  | |  | | | |  | | | | |  | | |  | | | |  |
| 23 | I suppose that offering CCS during pregnancy, will increase general participation significantly | | | |  | |  | | | |  | | | | |  | | |  | | | |  |
| **Negative statements on antenatal CCS** | | | | | | | | | | | | | | | | | | | | | | | |
|  |  | | Totally disagree | | | Disagree | | | | Neutral | | | | | Agree | | | Totally agree | | | | Totally disagree | |
| 24 | I think that pregnant women are offered to many screening already | |  | | |  | | | |  | | | | |  | | |  | | | |  | |
| 25 | I suppose CCS during pregnancy will cause unnecessary anxiety in many women | |  | | |  | | | |  | | | | |  | | |  | | | |  | |
| 26 | I suppose that CCS during pregnancy will lead to unnecessary referrals to second or third-line obstetric care | |  | | |  | | | |  | | | | |  | | |  | | | |  | |
| 27 | I think cervical cancer screening may be harmful for pregnancy | |  | | |  | | | |  | | | | |  | | |  | | | |  | |
| 28 | I consider offering cervical cancer screening no relevant task for obstetric care provider | |  | | |  | | | |  | | | | |  | | |  | | | |  | |
| 29 | I think that offering cervical cancer screening to pregnant women will cost me too much time | |  | | |  | | | |  | | | | |  | | |  | | | |  | |
| 30 | I suppose that general non-responders of CCS, will definitely not attend during pregnancy | |  | | |  | | | |  | | | | |  | | |  | | | |  | |
| **Statements related to previously answered questions** | | | | | | | | | | | | | | | | | | | | | | | |
| If performing colposcopy on a weekly base | | | | | | | | | | | | | | | | | | | | | | | |
|  |  | | | Totally disagree | | | | Disagree | | | | | | Neutral | | | Agree | | | | Totally agree | | Totally disagree |
| 31 | I tend to think that CCS during pregnancy is still to inaccurate, even after introduction of hrHPV-based screening | | |  | | | |  | | | | | |  | | |  | | | |  | |  |
| If performing colposcopy in pregnant women often | | | | | | | | | | | | | | | | | | | | | | | |
| 32 | I find colposcopy more difficult to interpret in pregnant women, compared to non-pregnant women | | |  | | | |  | | | | | |  | | |  | | | |  | |  |
