# Supplementary material for: Perspective of obstetric care‐providers on being involved in cervical cancer screening during antenatal care in the Netherlands
Source: Cancer Med. 2024 Jul 5;13(13):e7380. doi: 10.1002/cam4.7380 (PMC11224965; doi:10.1002/cam4.7380)
Supplement: Supplementary file 3 — Appendix S3. [file CAM4-13-e7380-s006.docx]

**Appendix C:** questionnaire provided to GPs and residents

| **Personal information** | | | | | | | | | | | | | | | | | | | | | |
| --- | --- | --- | --- | --- | --- | --- | --- | --- | --- | --- | --- | --- | --- | --- | --- | --- | --- | --- | --- | --- | --- |
| 1 | Current age | | … years | | | | | | | | | | | | | | | | | | |
| 2 | Are you employed currently? | |  Yes, as a resident in general practitioner   Yes, as a general practitioner   Yes, but not as a general practitioner, nor as a midwife   No | | | | | | | | | | | | | | | | | | |
| 3 | In which province(s) are you working currently? | |  Drenthe   Flevoland   Friesland   Gelderland | | | | | | |  Groningen   Limburg   Noord-Brabant   Noord-Holland | | | | | | | |  Overijssel   Utrecht   Zeeland   Zuid-Holland | | | |
| 4 | First-line practice located in | |  Small township (< 25.000 inhabitants)   Small city (25.000 – 100.000 inhabitants)   Major city (> 100.000 inhabitants) | | | | | | | | | | | | | | | | | | |
| 5 | Number of college’s in first-line midwifery practice | |  None   One   Two or more | | | | | | | | | | | | | | | | | | |
| 6 | Number of pregnant women per first-line midwifery practice | |  Less than 2000   2000 – 2999   3000 – 3999   More than 4000 | | | | | | | | | | | | | | | | | | |
| **Your experience with obstetric care** | | | | | | | | | | | | | | | | | | | | | |
|  |  | | | Totally disagree | | | Disagree | | | | | Neutral | | | Agree | | | | Totally agree | | |
| 7 | I provide obstetric care on a weekly base | | |  | | |  | | | | |  | | |  | | | |  | | |
| 8 | I always question CCS participation at pregnancy intake | | |  | | |  | | | | |  | | |  | | | |  | | |
| 9 | I always provide information on CCS to stated non-responders | | |  | | |  | | | | |  | | |  | | | |  | | |
| 10 | Pregnant women ask me whether I can perform CCS during pregnancy | | |   *Please go to question 12* | | |   *Please go to question 12* | | | | |   *Please go to question 12* | | |  | | | |  | | |
| 11 | I perform CCS, if pregnant women ask me | | |  | | |  | | | | |  | | |  | | | |  | | |
| 12 | Non-pregnant women ask me whether I can perform CCS | | |   *Please go to question 14* | | |   *Please go to question 14* | | | | |   *Please go to question 14* | | |  | | | |  | | |
| 13 | I perform CCS, if non-pregnant women ask me | | |  | | |  | | | | |  | | |  | | | |  | | |
| **Positive statements on antenatal CCS** | | | | | | | | | | | | | | | | | | | | | |
|  |  | Totally disagree | | | | Disagree | | | Neutral | | | | Agree | | | Totally agree | | | | Totally disagree | |
| 14 | I consider CCS during pregnancy via attended obstetric care provider feasible |  | | | |  | | |  | | | |  | | |  | | | |  | |
| 15 | I consider CCS during pregnancy via general practitioner feasible |  | | | |  | | |  | | | |  | | |  | | | |  | |
| 16 | I consider CCS of non-pregnant women via first-line midwives feasible |  | | | |  | | |  | | | |  | | |  | | | |  | |
| 17 | I suppose CCS is more easy to schedule during pregnancy, for a majority of women |  | | | |  | | |  | | | |  | | |  | | | |  | |
| 18 | I suppose women can be motivated more to attend CCS by obstetric care provider in person, rather than via anonymous leaflets |  | | | |  | | |  | | | |  | | |  | | | |  | |
| 19 | I suppose women are less encumbered by cervical sampling if performed by obstetric care provider rather than general practitioner |  | | | |  | | |  | | | |  | | |  | | | |  | |
| 20 | I think that most first-line midwives are skilled in performing cervical sampling |  | | | |  | | |  | | | |  | | |  | | | |  | |
| 21 | I think that most first-line midwives are well known with current CCS-programme |  | | | |  | | |  | | | |  | | |  | | | |  | |
| 22 | I suppose that offering CCS during pregnancy, will increase general participation significantly |  | | | |  | | |  | | | |  | | |  | | | |  | |
| **Negative statements on antenatal CCS** | | | | | | | | | | | | | | | | | | | | | |
|  |  | | | | Totally disagree | | | Disagree | | | Neutral | | | Agree | | | Totally agree | | | | Totally disagree |
| 23 | I think that pregnant women are offered to many screening already | | | |  | | |  | | |  | | |  | | |  | | | |  |
| 24 | I suppose CCS during pregnancy will cause unnecessary anxiety in many women | | | |  | | |  | | |  | | |  | | |  | | | |  |
| 25 | I suppose that CCS during pregnancy will lead to unnecessary referrals to second or third-line obstetric care | | | |  | | |  | | |  | | |  | | |  | | | |  |
| 26 | I think cervical cancer screening may be harmful for pregnancy | | | |  | | |  | | |  | | |  | | |  | | | |  |
| 27 | I consider offering cervical cancer screening no relevant task for obstetric care provider | | | |  | | |  | | |  | | |  | | |  | | | |  |
| 28 | I think that offering cervical cancer screening to pregnant women will cost me too much time | | | |  | | |  | | |  | | |  | | |  | | | |  |
| 29 | I suppose that general non-responders of CCS, will definitely not attend during pregnancy | | | |  | | |  | | |  | | |  | | |  | | | |  |
