# Supplementary material for: Perspective of obstetric care‐providers on being involved in cervical cancer screening during antenatal care in the Netherlands
Source: Cancer Med. 2024 Jul 5;13(13):e7380. doi: 10.1002/cam4.7380 (PMC11224965; doi:10.1002/cam4.7380)
Supplement: Supplementary file 4 — Appendix S4. [file CAM4-13-e7380-s005.docx]

**Appendix D:** supplementary personal and professional characteristics of respondents

|  | **General Practitioner** | **Gynecologist** | **Midwife** |
| --- | --- | --- | --- |
| ***Affiliation†*** | | | |
| Respondents (N) | 312 | 297 | 660 |
| First-line practice (%) | 100 | n.a. | 98.4 |
| General hospital (%) | - | 68.0 | 1.4 |
| University hospital (%) | - | 26.6 | 0.2 |
| Private clinic (%) | - | 5.4 | - |
| ***Province of employment†*** | | | |
| Respondents (N) | 314 | 289 | 700 |
| Drenthe (%) | 1.6 | 1.7 | 2.9 |
| Flevoland (%) | None | 1.4 | 1.7 |
| Friesland (%) | 2.9 | 2.1 | 4.3 |
| Gelderland (%) | 6.1 | 10.4 | 13.7 |
| Groningen (%) | 1.0 | 6.9 | 3.1 |
| Limburg (%) | 4.1 | 6.2 | 6.7 |
| Noord-Brabant (%) | 19.7 | 13.8 | 14.6 |
| Noord-Holland (%) | 5.1 | 21.8 | 15.4 |
| Overijssel (%) | 23.6 | 6.6 | 7.4 |
| Utrecht (%) | 5.4 | 5.9 | 7.9 |
| Zeeland (%) | 2.2 | 1.4 | 2.3 |
| Zuid-Holland (%) | 28.3 | 21.8 | 20.0 |
| ***First-line practice located in†*** | | | |
| Respondents (N) | 312 | n.a. | 698 |
| Small township with < 25.000 inhabitants (%) | 41.0 | n.a. | 38.0 |
| Small city with 25.000 – 100.000 inhabitants (%) | 28.2 | n.a. | 36.1 |
| Major city with > 100.000 inhabitants (%) | 30.8 | n.a. | 25.9 |
| ***Number of colleagues per first-line practice†*** | | | |
| Respondents (N) | 312 | n.a. | 651 |
| None (%) | 27.6 | n.a. | 6.0 |
| One (%) | 29.8 | n.a. | 13.4 |
| Two or more (%) | 42.6 | n.a. | 80.6 |
| ***Number of patients per GPs practice†*** | | | |
| Respondents (N) | 310 | n.a. | n.a. |
| Less than 2000 (%) | 4.5 | n.a. | n.a. |
| 2000 to 2999 (%) | 37.4 | n.a. | n.a. |
| 3000 to 3999 (%) | 18.4 | n.a. | n.a. |
| 4000 or more (%) | 39.7 | n.a. | n.a. |
| ***Number of pregnant women per first-line midwifery practice†*** | | | |
| Respondents (N) | n.a. | n.a. | 622 |
| Less than 100 (%) | n.a. | n.a. | 0.2 |
| 100 to 299 (%) | n.a. | n.a. | 54.3 |
| 300 to 499 (%) | n.a. | n.a. | 36.8 |
| 500 or more (%) | n.a. | n.a. | 8.7 |
| ***Professional scope as gynecologists or resident†*** | | | |
| Respondents (N) | 378 | n.a. | n.a. |
| Perinatology (%) | 24.6 | n.a. | n.a. |
| Oncology (%) | 20.1 | n.a. | n.a. |
| Urogynecology (%) | 14.3 | n.a. | n.a. |
| Benign gynecology (%) | 29.9 | n.a. | n.a. |
| Reproductive medicine (%) | 11.1% | n.a. | n.a. |

†Multiple answers possible
