# Supplementary material for: Perspective of obstetric care‐providers on being involved in cervical cancer screening during antenatal care in the Netherlands
Source: Cancer Med. 2024 Jul 5;13(13):e7380. doi: 10.1002/cam4.7380 (PMC11224965; doi:10.1002/cam4.7380)
Supplement: Supplementary file 5 — Appendix S5. [file CAM4-13-e7380-s003.docx]

**Appendix E:** Answers to favorable statements regarding antenatal CCS by profession

|  | **General Practitioners** | **Gynecologists** | **Midwifes** | **Total** |
| --- | --- | --- | --- | --- |
| ***I consider CCS during pregnancy via general practitioner feasible*** | | | | |
| Respondents (*N)* | 301 | 284 | 641 | 1226 |
| Agree (%) | 70.8 | 77.1 | 65.6 | 69.6 |
| Neutral (%) | 16.7 | 11.3 | 20.8 | 17.6 |
| Disagree (%) | 8.5 | 11.0 | 12.2 | 11.9 |
| No opinion (%) | 3.9 | 0.7 | 1.4 | 1.9 |
| *Chi-square test: GPs vs gynecologists p = 0.01; GPs vs midwifes p = 0.01; gynecologists vs midwifes p = 0.002* | | | | |
| ***I suppose CCS is more easy to schedule during pregnancy, for a majority of women*** | | | | |
| Respondents (*N)* | 294 | 285 | 638 | 1217 |
| Agree (%) | 40.1 | 50.5 | 49.5 | 47.5 |
| Neutral (%) | 22.8 | 24.6 | 23.0 | 23.3 |
| Disagree (%) | 35.4 | 24.9 | 26.5 | 28.3 |
| No opinion (%) | 1.7 | None | 0.9 | 0.3 |
| *Chi-square test: GPs vs gynecologists; p = 0.003 ; GPs vs midwifes p = 0.17; gynecologists vs midwifes p = 0.38* | | | | |
| ***I suppose women can be motivated more to attend CCS by obstetric care provider in person, rather than via anonymous leaflets*** | | | | |
| Total (*N)* | 293 | 288 | 638 | 1222 |
| Agree (%) | 74.4 | 83.7 | 90.6 | 85.1 |
| Neutral (%) | 11.6 | 7.6 | 5.6 | 7.5 |
| Disagree (%) | 13.7 | 8.7 | 3.3 | 7.0 |
| No opinion (%) | 0.3 | None | 0.5 | 0.3 |
| *Chi-square test: GPs vs gynecologists; p = 0.04 ; GPs vs midwifes p < 0.001 ; Gynecologists vs midwifes p = 0.001* | | | | |
| ***I suppose women are less encumbered by cervical sampling if performed by obstetric care provider rather than general practitioner*** | | | | |
| Respondents (*N)* | 294 | 287 | 638 | 1219 |
| Agree (%) | 25.9 | 46.3 | 92.2 | 65.4 |
| Neutral (%) | 23.1 | 26.1 | 6.1 | 14.9 |
| Disagree (%) | 49.3 | 25.4 | 1.7 | 18.8 |
| No opinion (%) | 1.7 | 2.1 | None | 0.9 |
| *Chi-square test: GPs vs gynecologists; p < 0,001 ; GPs vs midwifes p < 0,001 ; Gynecologists vs midwifes p < 0,001* | | | | |
| ***I suppose that offering CCS during pregnancy, will increase general participation significantly*** | | | | |
| Respondents (*N)* | 294 | 287 | 642 | 1223 |
| Agree (%) | 45.6 | 24.4 | 70.4 | 53.6 |
| Neutral (%) | 28.9 | 16.7 | 17.6 | 20.1 |
| Disagree (%) | 23.1 | 58.9 | 9.5 | 24.4 |
| No opinion (%) | 2.4 | None | 2.5 | 1.9 |
| *Chi-square test: GPs vs gynecologists; p < 0.001 ; GPs vs midwifes p < 0.001; Gynecologists vs midwifes p < 0.001* | | | | |
| ***I always question CCS participation at pregnancy intake†*** | | | | |
| Respondents (*N)* | None | 118 | 647 | 765 |
| Agree (%) | - | 14.4 | 82.5 | 72.0 |
| Neutral (%) | - | 12.7 | 2.2 | 3.8 |
| Disagree (%) | - | 71.2 | 15.1 | 23.8 |
| No opinion (%) | - | 1.7 | None | 0.4 |
| *Chi-square test: gynecologists vs midwifes p < 0.001* | | | | |
| ***I always provide information on CCS to stated non-responders‡*** | | | | |
| Respondents (*N)* | None | 17 | 532 | 550 |
| Agree (%) | - | 64.7 | 68.0 | 67.8 |
| Neutral (%) | - | 17.6 | 11.1 | 11.3 |
| Disagree (%) | - | 17.6 | 20.5 | 20.5 |
| No opinion (%) | - | None | 0.4 | 0.4 |
| *Chi-square test: gynecologists vs Midwifes p = 0.85* | | | | |
| ***Pregnant women ask me whether I can perform CCS during pregnancy†*** | | | | |
| Respondents (*N)* | None | 118 | 643 | 761 |
| Agree (%) | - | 7.6 | 25.5 | 22.7 |
| Neutral (%) | - | 7.6 | 10.3 | 9.9 |
| Disagree (%) | - | 84.7 | 64.1 | 67.3 |
| No opinion (%) | - | None | 0.2 | 0.1 |
| *Chi-square test: gynecologists vs midwifes p < 0.001* | | | | |
| ***I consider myself skilled in performing cervical sampling (%) §*** | | | | |
| Respondents (*N)* | n.a. | n.a. | 654 | n.a. |
| Agree (%) | n.a. | n.a. | 49.7 | n.a. |
| Neutral (%) | n.a | n.a | 10.3 | n.a |
| Disagree (%) | n.a. | n.a. | 38.9 | n.a. |
| No opinion (%) | n.a. | n.a. | 1.1 | n.a. |
| ***I consider myself familiar with current Dutch CCS programme (%) §*** | | | | |
| Respondents (*N)* | n.a. | n.a. | 641 | n.a. |
| Agree (%) | n.a. | n.a. | 47.0 | n.a. |
| Neutral (%) | n.a | n.a | 28.5 | n.a |
| Disagree (%) | n.a. | n.a. | 23.9 | n.a. |
| No opinion (%) | n.a. | n.a. | 0.6 | n.a. |

†This question was only presented to those respondents stating to provide obstetric care weekly

‡This question was only presented to those respondents answering the statement *‘I always question CCS participation at pregnancy intake’* either with *‘*agree’ or *‘*totally agree’.

§These questions were only presented to midwifes.
