# Supplementary material for: Perspective of obstetric care‐providers on being involved in cervical cancer screening during antenatal care in the Netherlands
Source: Cancer Med. 2024 Jul 5;13(13):e7380. doi: 10.1002/cam4.7380 (PMC11224965; doi:10.1002/cam4.7380)
Supplement: Supplementary file 6 — Appendix S6. [file CAM4-13-e7380-s001.docx]

**Appendix F**: provided answers to unfavorable statements regarding antenatal CCS.

|  | | **General practitioners** | **Gynecologists** | **Midwives** | **Total** |
| --- | --- | --- | --- | --- | --- |
| ***I think that pregnant women are offered too many screening already*** | | | | | |
| Respondents (*N)* | 295 | | 286 | 641 | 1222 |
| Agree (%) | 11.2 | | 36.7 | 17.8 | 20.6 |
| Neutral (%) | 25.4 | | 22.0 | 34.3 | 29.3 |
| Disagree (%) | 61.0 | | 40.9 | 46.8 | 48.9 |
| No opinion (%) | 2.4 | | 0.3 | 1.1 | 1.2 |
| *Chi-square test: GPs vs Gynecologists p < 0.001; GPs vs Midwifes p < 0.001; Gynecologists vs Midwifes p < 0.001;* | | | | | |
| ***I suppose CCS during pregnancy will cause unnecessary anxiety in many women*** | | | | | |
| Respondents (*N)* | 295 | | 287 | 636 | 1218 |
| Agree (%) | 33.9 | | 29.6 | 34.3 | 33.1 |
| Neutral (%) | 23.1 | | 15.0 | 31.1 | 25.5 |
| Disagree (%) | 41.0 | | 55.4 | 32.2 | 39.8 |
| No opinion (%) | 2.0 | | None | 2.2 | 1.6 |
| *Chi-square test: GPs vs Gynecologists p < 0.001; GPs vs Midwifes p = 0.26; Gynecologists vs Midwifes p = 0.85* | | | | | |
| ***I suppose that CCS during pregnancy will lead to unnecessary referrals to second or third-line obstetric care*** | | | | | |
| Respondents (*N)* | 294 | | 284 | 640 | 1218 |
| Agree (%) | 27.2 | | 62.7 | 29.1 | 36.5 |
| Neutral (%) | 22.8 | | 22.9 | 33.1 | 28.2 |
| Disagree (%) | 43.2 | | 12.0 | 32.2 | 30.1 |
| No opinion (%) | 6.8 | | 2.5 | 5.6 | 5.2 |
| *Chi-square test: GPs vs Gynecologists p < 0.001; GPs vs Midwifes p = 0.002; Gynecologists vs Midwifes p < 0,001* | | | | | |
| ***I think cervical cancer screening may be harmful for pregnancy*** | | | | | |
| Respondents (*N)* |  | 295 | 288 | 638 | 1221 |
| Agree (%) | 5.4 | | 5.6 | 5.6 | 5.6 |
| Neutral (%) | 10.8 | | 8.3 | 16.5 | 13.0 |
| Disagree (%) | 80.0 | | 85.8 | 73.0 | 77.7 |
| No opinion (%) | 4.4 | | 0.3 | 0.9 | 3.7 |
| *Chi-square test: GPs vs Gynecologists; p = 0.11 ; GPs vs Midwifes p = 0.08; Gynecologists vs Midwifes p = 0.38* | | | | | |
| ***I consider offering cervical cancer screening no relevant task for obstetric care provider*** | | | | | |
| Total (*N)* | 295 | | 287 | 638 | 1218 |
| Agree (%) | 39.7 | | 26.5 | 12.3 | 22.2 |
| Neutral (%) | 24.4 | | 19.9 | 18.9 | 20.4 |
| Disagree (%) | 33.2 | | 52.6 | 67.9 | 55.9 |
| No opinion (%) | 2.7 | | 1.0 | 0.9 | 1.4 |
| *Chi-square test: GPs vs Gynecologists; p < 0.001 ; GPs vs Midwifes p < 0.001 ; Gynecologists vs Midwifes p = 0.001* | | | | | |
| ***I suppose that general non-responders of CCS, will definitely not attend during pregnancy*** | | | | | |
| Respondents (*N)* | 293 | | 115 | 639 | 1047 |
| Agree (%) | 37.2 | | 20.0 | 34.0 | 33.3 |
| Neutral (%) | 21.8 | | 21.7 | 24.7 | 23.6 |
| Disagree (%) | 36.5 | | 58.3 | 37.9 | 39.7 |
| No opinion (%) | 4.4 | | 0 | 3.4 | 3.3 |
| *Chi-square test: GPs vs Gynecologists; p < 0.001 ; GPs vs Midwifes p = 0.587 ; Gynecologists vs Midwifes p < 0.001* | | | | | |
| ***I think that offering cervical cancer screening to pregnant women will cost me too much time*** | | | | | |
| Respondents (*N)* | 14 | | 287 | 641 | 942 |
| Agreee (%) | None | | 24.4 | 24.2 | 23.9 |
| Neutral (%) | 21.4 | | 25.1 | 25.9 | 25.6 |
| Disagree (%) | 78.6 | | 47.4 | 48.5 | 48.6 |
| No opinion (%) | None | | 3.1 | 1.4 | 1.9 |
| *Chi-square test: GPs vs Gynecologists; p = 0.09 ; GPs vs Midwifes p = 0.102; Gynecologists vs Midwifes p < 0.001* | | | | | |
